# Hard to Take

**Published:** 1869-12

**Authors:** 


### HARD TO TAKE.

A LADY correspondent, a former patient, writes: "Allow me to thank you for your kind, common-sense advice, through whatever channel it may have come. Your Journal is prized by us, and read with much pleasure. I wish it might be taken by every family in the land." We certainly respond with great cordiality to the wishes of our correspondent, for the good which we know it would accomplish, irrespective of any advantage to ourself. In obtaining a husband, this lady drew a double prize, first, in getting one who is "well to do" in a pecuniary point of view, and unusually considerate of his wife's comfort besides; for we very well know that there are a good many supremely selfish husbands in the world. He rises betimes, and remains in his counting-room till dark, and coming home, almost too tired to eat, still remains an hour or two until his wife can get ready, that he may carry the baby up stairs, it going to sleep regularly at dark. Can't say for ourselves that we think our correspondent does exactly the right thing, in taking two hours every night to get ready to retire.

Politeness, and acts of consideration, deference, and kindness usually provoke a retaliation in kind. What a handsome young woman can possibly be doing for two mortal hours, in every twenty-four, in order to get ready for rest, we cannot possibly conceive. If she wore a wig, and had false teeth, and needed the aid of divers pounds of cotton batting to make things look proportionate, to say nothing of stays to keep her straight, and "abominable supporters" to keep her "up," such a time might be required; but our correspondent being young and handsome, and of form and mein needing no adventitious aids, we rather think she must be chargeable with inconsideration. She then goes on to complain of two very considerable inconveniences, which her own common sense attributes to being "excited and very tired."

Now, it so happens that we have an admirable remedy for the removal of these very prevalent causes of much of the ill-health and miserableness of many of the wives of this land—causes which make married life a blight instead of a blessing, and which make half of that "motherless" army which may any day be martialed, and record the "hard knocks" along life's pathway, which it is their destiny to endure, without any hope of amelioration, until with gladness they leave the roof which had been to them no shelter and no home, since "mother's" death.

Unfortunately, our medicine is harder to take than assafetida, castor oil, or corn cobs. Three times out of four the avowal is frankly made: "I can't take it." But whoever heard of a woman failing to do what she wanted—what she had set her heart upon? To any femininity on the globe, "can't" is the utterist figment of the most aerial imagination. If you respond, why can't you? they repeat the same old sing-song, stereotyped reply—the same everlasting refrain—which leaped out, ready made, the day Eve took her first sewing lesson: "I've so much sewing to do." The slowest Jonathan may easily calculate where all the pins go to, and what becomes of all the Yankee clocks; but we defy a regiment of the 'cutest Connecticutcheons to "guess" where all the "sewing" is stowed away which women have been at, real or imaginary since first "dress" has come into fashion. A reasonable man would think that everything on the face of the earth that could be "seamed," or sewn together, would have been done long ago, and that no more needles need be made for the next generation. But strange as it may seem, our wives are right, the sewing is never done. We have never yet been in a household where some was not wanted—a very little job which we do not remember of late to have seen thoroughly done, if ever, although a very few stitches would suffice. But women are self-sacrificing, and do for the children and husband first, while themselves are neglected. The job we speak of is very generally done for the husband immediately after marriage, if not sooner; while the wife seldom finds time to do it for herself until he, "poor man," is under the sod, when the widow's lips are so closed to all his faults and failings, that you would not know that he was not one of the most perfect of men. Her lips are sewed now. How many a good fellow, now no more, might have been still walking on the earth, in health and gladness, if, on the day of marriage, the first act after the ceremony had been to sew the new wife's lips together! Now that the sewing machine has become a domestic institution, and has become woman's deliverer from a thraldom under which she has groaned and ground out her life, we trust that it will no longer be said, as an excuse for late hours, and over work, and ruinous confinement to in-doors: "There is so much sewing to do."

Our correspondent complained of being easily "excited, and becoming very tired." We simply advised her to take things easy. No doubt, her considerate husband had given the same advice dozens of times, and most likely the reply had as often been made: "I can't;" "How can, I when there is so much to do?" and away goes the wife again, from cellar to garret, and works as if her life depended on it all being properly done in a given time—the result being, that, overheated and perfectly exhausted, she flings herself on a bed, falls to sleep, cools off rapidly, wakes up, feels chilly and bad all over. A cold has been taken, and she is fit for nothing, until it has run its course of about two weeks, with, most likely, a doctor's bill intervening, which would more than pay for all the work of a year. Let us ask any considerate wife just here, would it not have been better to have taken three days to do that work, and continued well, than to have done it in a few hours, and lost two weeks in sickness, to say nothing of the extra work and inconvenience which it necessarily entailed on every member of the family? Yet, this is literally the way in which many of our wives act. They do so with their eyes open, and against the known wishes and earnest remonstrance of their husbands. It is useless to mince matters of this kind, and we desire to say plainly, that wives who thus act, do so under the influence of a stubborn folly, which is alike disgraceful to their intelligence and their moral nature.

